# Longitudinal Trajectories in Cortical Thickness and Volume Atrophy: Superior Cognitive Performance Does Not Protect Against Brain Atrophy in Older Adults

**DOI:** 10.3233/JAD-201243

**Published:** 2021-06-01

**Authors:** Samantha L. Gardener, Michael Weinborn, Hamid R. Sohrabi, James D. Doecke, Pierrick Bourgeat, Stephanie R. Rainey-Smith, Kai-kai Shen, Jurgen Fripp, Kevin Taddei, Paul Maruff, Olivier Salvado, Greg Savage, David Ames, Colin L. Masters, Christopher C. Rowe, Ralph N. Martins, for the AIBL Research Group

**Affiliations:** a Centre of Excellence for Alzheimer’s Disease Research & Care, School of Medical and Health Sciences, Edith Cowan University, Joondalup, Western Australia, Australia; b Australian Alzheimer’s Research Foundation, Perth, Western Australia, Australia; c School of Psychological Science, University of Western Australia, Crawley, Western Australia, Australia; d College of Science, Health, Engineering and Education, Murdoch University, Murdoch, Western Australia, Australia; e Department of Biomedical Sciences, Macquarie University, New South Wales, Australia; f CSIRO Health and Biosecurity/Australian eHealth Research Centre, Herston, Queensland, Australia; g Centre for Healthy Ageing, Health Futures Institute, Murdoch University, Murdoch, Western Australia, Australia; h CogState, Ltd., Melbourne, Victoria, Australia; i CSIRO Data61, Sydney, Australia; j ARC Centre of Excellence in Cognition and its Disorders and Department of Psychology, Macquarie University, New South Wales, Australia; k National Ageing Research Institute, Royal Melbourne Hospital, Melbourne, Australia; l Academic Unit for Psychiatry of Old Age, University of Melbourne, Melbourne, Australia; m The Florey Institute of Neuroscience and Mental Health, The University of Melbourne, Parkville, Victoria, Australia; n Department of Molecular Imaging and Therapy, Centre for PET, Austin Health, Heidelberg, Victoria, Australia; o Florey Department of the University of Melbourne; p http://www.aibl.csiro.au (for the AIBL Research Group)

**Keywords:** Cognitive aging, cortical thickness, cortical thinning, cerebral volume atrophy, older adult superior cognitive performance, super-aging

## Abstract

**Background:**

Previous research has identified a small subgroup of older adults that maintain a high level of cognitive functioning well into advanced age. Investigation of those with superior cognitive performance (SCP) for their age is important, as age-related decline has previously been thought to be inevitable.

**Objective:**

Preservation of cortical thickness and volume was evaluated in 76 older adults with SCP and 100 typical older adults (TOAs) assessed up to five times over six years.

**Methods:**

Regions of interest (ROIs) found to have been associated with super-aging status (a construct similar to SCP status) in previous literature were investigated, followed by a discovery phase analyses of additional regions. SCPs were aged 70 + at baseline, scoring at/above normative memory (CVLT-II) levels for demographically similar individuals aged 30–44 years old, and in the unimpaired range for all other cognitive domains over the course of the study.

**Results:**

In linear mixed models, following adjustment for multiple comparisons, there were no significant differences between rates of thinning or volume atrophy between SCPs and TOAs in previously identified ROIs, or the discovery phase analyses. With only amyloid-β negative individuals in the analyses, again there were no significant differences between SCPs and TOAs.

**Conclusion:**

The increased methodological rigor in classifying groups, together with the influence of cognitive reserve, are discussed as potential factors accounting for our findings as compared to the extant literature on those with superior cognitive performance for their age.

## INTRODUCTION

The majority of older adults experience some decline in aspects of cognitive functions with increasing age, including episodic memory, speed of processing, and executive functions. However, previous research [1–5] has identified a small subgroup of older adults that maintain a high level of cognitive functioning well into advanced age. The constructs used to describe such superior cognitive aging (e.g., “super-aging”) have utilized overlapping but somewhat different criteria selected by different researchers (e.g., minimum age criteria, or cognitive domain assessed). However, all appear to have agreed that this construct requires an individual to perform cognitively at a level similar to those at least 20 to 30 years younger than their peer group. Investigation of those with superior cognitive performance (SCP) for their age is important, as age-related decline has previously been thought to be inevitable. Differentiating the changes intrinsic to aging, from those that are common but may be preventable, for example lifestyle factors, is becoming one of the most important goals of aging research.

Table 1 presents the areas previously found to have increased cortical thickness and volumes in older adults with SCP compared to typical older adults (TOAs; that is older adults that show the expected age-related declines in cognitive function over time) in the extant literature.

**Table 1:** Areas found to have preserved cortical thickness and volume in published studies of individuals with superior cognitive performance

Title, Author, Year	Number of participants	Domain used to define SCP	Volume or Thickness	Areas
Selective Increase of Cortical thickness in high-performing elderly-structural indices of optimal cognitive aging.[2]	39 old (mean age 70.7, SD 7.0)	Executive function, and fluid function	Thickness	Posterior parts of cingulate gyrus –right hemisphere
	35 young (mean age 35.5, SD 0.8)			Some frontal and prefrontal areas in both hemispheres
	Median split to divide into high and low fluid performers			Medial structure and gyrus of the cingulate isthmus
Superior memory and higher cortical volumes in unusually successful cognitive aging [1]	12 SA (mean age 83.5, SD 3.0)	Episodic memory	Volume	Global volume
	10 TOA (mean age 83.1, SD 3.4)		Thickness	Global cortex
	14 middle aged (mean age 57.9, SD 4.3)			Left anterior cingulate cortex
Morphometric and histologic substrates of cingulate integrity in elders with exceptional memory capacity [4]	31 SA (mean age 82.52, SD 2.93)	Episodic memory	Thickness	Right rostral anterior cingulate Posterior cingulate Caudal anterior regions
	21 TOA (mean age 83.76, SD 4.0)
	18 middle aged (mean age 58.39, SD 3.7)
Youthful brains in older adults: preserved neuroanatomy in the default mode and salience networks contributes to youthful memory in super-aging [3]	17 SA (mean age 67.8, SD 6.0)	Episodic memory	Volume	Right hippocampus
	23 TOA (mean age 66.2, SD 5.1)		Thickness	Right angular gyrus
	41 young adult (mean age 24.5, SD 3.6)			Right superior frontal gyrus
				Left anterior middle temporal gyrus
				Bilateral rostral medial prefrontal cortex
				Left dorsomedial prefrontal cortex
				Bilateral midcingulate cortex
				Left midinsula
				Right dorsal anterior insula
				Right frontal operculum
				Right dorsolateral prefrontal cortex
				Right inferior frontal gyrus
				Left primary somatosensory correct
				Left lateral occipital cortex
				Calcarine cortical regions 1 and 2
Rates of Cortical Atrophy in Adults 80 Years and Older With Superior vs Average Episodic Memory [7]	24 SA (mean age 83.3, SD 3.5), 12 cognitively average elderly adults (mean age 83.4, SD 3.8)	Episodic memory	Volume	Whole brain cortical volume
Brain Morphology, cognition, and Aβ in older adults with superior memory performance [6]	26 successful agers (mean age 74.9, SD 4.6)	Episodic memory	Volume Thickness	Hippocampal Right anterior cingulate Prefrontal cortex
	103 TOA (mean age 75.9, SD 4.5)
Rates of age- and amyloid β-associated cortical atrophy in older adults with superior memory performance [9]	172 SA, 172 Cognitively normal for age (mean age 71.75, median age 71.00)	Episodic memory	Volume	No significant associations observed in white matter, gray matter, hippocampus, and white matter hyperintensity

SA, super-ager; SD, standard deviation; TOA, typical older adult.

### Cross-sectional studies

Fjell et al. [2] were the first group to discuss this general SCP construct and described such individuals as ‘high fluid performers’ based on performance on fluid reasoning and executive functioning tests. Specifically, the authors split 74 participants (20–88 years of age) into high and low age groups, and further divided each age group into high and average performers depending on results of these fluid ability measures. They observed participants in the high age group/high fluid ability group had large areas of thicker cortex in comparison to the high age group/average fluid ability group. The largest differences were found in the posterior parts of the right cingulate gyrus. Additionally, differences were found in some frontal and prefrontal areas between groups in both hemispheres, as well as the medial structure and the gyrus of the cingulate isthmus.

Sun et al. [3] used a definition of super-agers that included individuals aged 60 and above (*n* = 17, mean age of 67.8 years) and compared them with a group of 23 TOAs (mean age of 66.2 years) and 41 young adults (mean age of 24.5 years). They found that SAs displayed significantly greater cortical thickness than TOAs, but equivalent to young adults in the anterior temporal cortex, rostral medial prefrontal cortex, and anterior mid-cingulate cortex. These are key paralimbic and limbic nodes of the default mode and salience networks usually engaged during attention, motivation, and executive function tasks.

Harrison et al. [1] initially identified a specific subset of older adults with SCP, described as “super agers” (SAs). The authors proposed a definition requiring an age of 80 or more, with episodic memory performance within or above the average normative values for 50–65-year-olds, and within one standard deviation of the average range for their age on non-memory measures particularly vulnerable to change in aging and dementia. They found 12 individuals meeting these SA criteria and compared them with 10 demographically matched healthy elderly peers, as well as 14 demographically matched middle-aged controls aged 50–65. They found the SAs had a region of left anterior cingulate cortex significantly thicker than both comparison groups. They additionally observed significantly larger cortical volume in the SAs compared to the same-aged peers, with no difference between SAs and the middle-aged group.

In a subsequent paper, Harrison and colleagues studied a similar SCP group of older adults, which included individuals aged 70 and above, described as “successful agers”. They compared 26 successful agers with 103 TOAs aged 70 and above. The authors observed greater cortical thickness in multiple regions including the right anterior cingulate and prefrontal cortex, and a greater hippocampal volume in successful agers than TOAs [6].

### Longitudinal studies

In addition to the cross-sectional analyses described above, Harrison et al. [6] followed 19 of the successful agers and 70 of the TOAs for at least one additional MRI scan (mean 1.54 additional scans) that allowed for observation of potential longitudinal decline in these regions up to approximately 3–5 years later for this small sub-sample. Results showed no difference in hippocampal volume atrophy rates, or whole cortex cortical thinning between successful agers and TOAs. While they observed that successful agers displayed slower cortical thinning in a number of left hemisphere areas including anterior cingulate, middle cingulate, medial prefrontal, and insula, there was no difference in rate of cortical thinning between successful agers and TOAs when these areas were combined bilaterally [6].

Cook et al. [7] found that 24 SAs (mean age 83.3 years) and 12 cognitively average elderly adults (mean age 83.4 years) demonstrated statistically significant mean annual percent whole-brain cortical volume loss over 18 months; however, the volume loss in the cognitively average elderly adults was significantly greater compared with the SAs. Both groups had similar levels of education and premorbid intellectual ability and had stable cognitive status across the 2 visits to minimize inclusion of individuals with emerging dementia.

Dang et al. [8] recently assessed longitudinal changes in global white matter, global grey matter, hippocampal regions, and white matter hyperintensity volumes over an 8-year follow-up in a cohort of 172 super-agers (using the aged 60 and above criteria) and 172 Cognitively Normal For Age (CNFA) individuals from the Australian Imaging Biomarkers and Lifestyle (AIBL) study of ageing, investigating the extent to which these rates are influenced by elevated amyloid-β (Aβ). The authors concluded the slowest rates of atrophy were observed in Aβ- participants, regardless of SA/CNFA status, and white matter hyperintensity volume increased at the same rate for all participants. Their results suggest that individuals classified as SA at baseline are not resilient to the neurobiological changes associated with age or Aβ deposition.

The previous literature has several limitations, and a multitude of questions remain unanswered. Longitudinal neuroimaging data is limited, for example Dang et al. [8] examined global grey matter volume but not cortical thickness or specific regional volume or thickness other than hippocampus. Harrison et al. [6] also examined only hippocampal volume with no specific regional volume analyses, and thickness of the whole cortex and only those cortical regions that showed greater thickness in successful agers in their cross-sectional analysis, and therefore trajectory of change in regional cortical thickness and cerebral volume over extended periods with an adequate sample size has not been thoroughly analyzed. It has been shown that cortical thinning occurs with advancing age [10], with global thinning apparent by middle age; however, it is currently unknown whether older adults with SCP experience similar rates of thinning as their cognitively average peers, or whether they resist these age-related changes. Secondly, from a methodological standpoint, previous studies have not established the stability of cognitive function used to classify older adults with SCP (regardless of age criteria) and TOAs. Specifically, previous studies relied on a psychometric classification of SCP versus TOA status, assessed on only a single occasion, leading to the potential for misclassification in both groups. A more reliable approach would be to confirm the presence of superior cognitive performance over multiple assessments, thereby increasing the reliability of SCP classification. Additionally, without longitudinal assessment it is impossible to determine whether those identified as TOAs may be in the early stages of cognitive decline, and therefore whether the SCPs are being compared to a TOA group that is truly aging typically and not containing individuals with preclinical dementia. Finally, the role of Aβ positivity in the trajectory of changes in cortical thickness and volume has not been evaluated across specific brain regions amongst older adults with SCP.

The current study evaluated longitudinal trajectories of brain morphology in 76 SCPs and 100 TOAs by quantitatively comparing preservation of cortical thickness and volume as measured by structural magnetic resonance imaging (MRI) scans. This investigation included a well-characterized, Australian older adult cohort drawn from the larger AIBL study [10]. Specifically, the current study aimed to evaluate whether the regions of interest (ROIs) identified in previous cross-sectional research as being relevant for superior cognitive performance (Table 1) show decreased cortical thinning and atrophy over time. In addition, we aimed to investigate other potentially relevant regions not identified in previous cross-sectional studies, but which may show such changes over time when analyzed longitudinally in a discovery phase analysis. The current study utilized a more stringent definition of SCP than previous studies. Specifically, participants in the SCP group had to meet the cognitive criteria over at least three consecutive time-points, therefore, stability of SCPs and TOAs cognitive ability and cognitively healthy status could be assessed in the classification of SCP and TOA. This allowed us to minimize inclusion of individuals with emerging cognitive decline. We hypothesized that SCPs would have reduced cortical thinning and atrophy over time compared to TOAs in the regions identified by the previous research (Table 1). We did not have specific hypotheses for the discovery phase analyses.

An important pathological process related to the development of cognitive decline and Alzheimer’s disease (AD) is Aβ deposition. The analyses were repeated following removal of participants above the accepted cut-off values for significant Aβ deposition and therefore on a pathway to AD as opposed to typical aging. This approach allowed the additional investigation of the role of Aβ status within the SCP construct.

Of note, a commentary by Rogalski [11] was published concurrently with the article by Dang et al. [8] raising concerns that the relatively younger age group used in the Dang et al. study (aged 60+) might explain the non-significant findings and recommending examining only SAs aged 80 + . It is notable that only one of the studies (Harrison et al, 2012 with only 12 SAs) reviewed above used this age cut-off, and the other relevant studies of super-aging or similar constructs/terms used lower age thresholds (e.g., aged 60 or 70 rather than 80+). This is likely due to the highly challenging nature of identifying such unusual individuals. To balance the concerns regarding an age at baseline that may be too low to allow detection of relationships of interest to the SA or older adult with SCP constructs, we used a criterion of aged 70 years or greater at baseline. We subsequently used a normative memory score for individuals aged 30–44 years rather than 50–65 years for classification as SCP. This allowed a sample of SCPs of sufficient size for longitudinal analysis which is lacking in the current literature. In addition, we included a cross-sectional *post-hoc* analysis in the subgroup of the current cohort aged 80 + . This *post-hoc* analysis utilized participants’ first MRI after they turned 80 years of age and investigated between group differences (SCPs versus TOAs) in ROIs identified in the literature among this age group.

## MATERIALS AND METHODS

### Participants

**Fig. 1:**
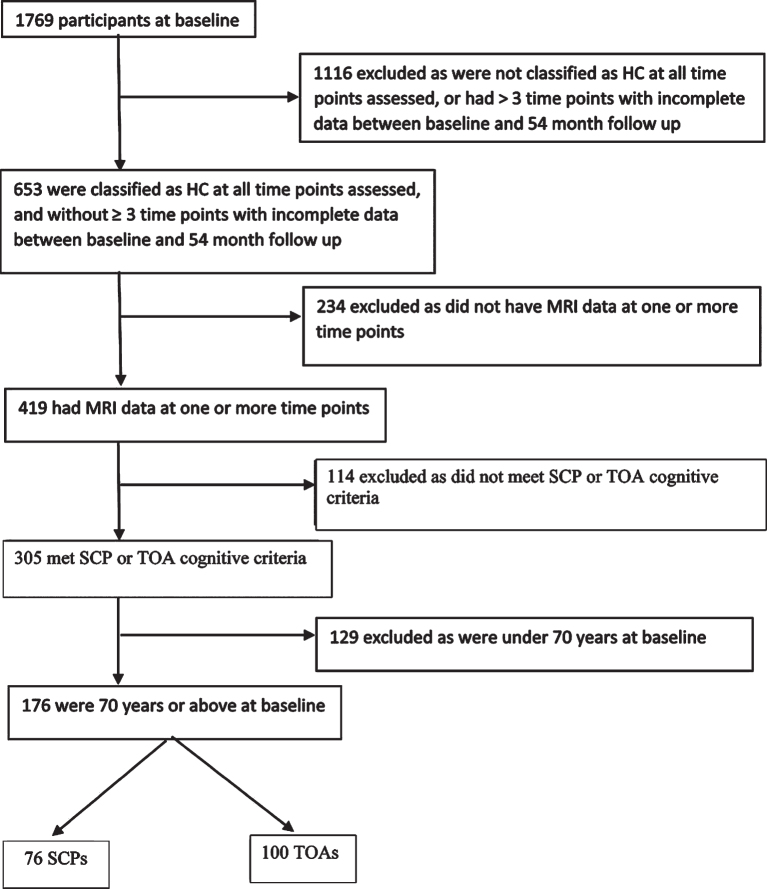
Flow chart for sample selection HC, healthy control; MRI, magnetic resonance imaging; SCP, superior cognitive performer; TOA, typical older adult.

This report describes data from 76 SCPs and 100 TOAs from the AIBL study [10]. Figure 1 shows the sample selection process. Methods of recruitment, assessment, inclusion and exclusion criteria for the AIBL study have been detailed previously, and the study has been approved by the institutional ethics committees of Austin Health, St Vincent’s Health, Hollywood Private Hospital and Edith Cowan University [10]. All participants were cognitively healthy based on a comprehensive battery of neuropsychological measures, including the Clinical Dementia Rating (CDR) scale and Mini-Mental State Examination (MMSE) score at four time points over 54 months (each 18 months apart). Participants completed brain MRI at one or more of five time points, and positron emission tomography (PET) imaging to quantify cerebral Aβ load was also completed at the corresponding time as the MRI. Of note, these participants were drawn from the same AIBL cohort as included in Dang et al. [8] study. However, our SCP and TOA classification requirements differ substantially from those used in that study. Therefore, the SCP and TOA groups in the present study consist of a subset of those used in the Dang et al. paper (i.e., 76 of their 172 SAs). Specifically, we have excluded all AIBL participants that did not meet our more stringent longitudinal SCP and TOA requirements.

### Cognitive assessments

A comprehensive neuropsychological battery of well-validated measures was administered according to standard protocols (described elsewhere [10, 12]). The battery assessed six cognitive domains (verbal and visual memory, executive function, language, attention and visuospatial functioning) at baseline, and 18, 36, and 54 months follow up where available.

### Defining older adult SCPs and TOAs

To define the SCP/TOA classification for analyses, participants were aged at least 70 at baseline, and were required to have completed the neuropsychological battery on at least three of the four time points (baseline, and 18, 36, and 54 months). SCPs must have, 1) attained at least normative mean memory (California Verbal Learning Test—Second Edition; CVLT-II) long-delayed free recall scores for individuals aged 30–44 (*z* scores ([value –mean]/standard deviation) no less than –0.5), and 2) normative scores in the unimpaired range (*z* above –1.5) for all other domains based on same-aged peers at all time points assessed. Use of long-delayed verbal memory scores has been common in previous research in this area [1, 6–8] as it is one of the more sensitive domains to age-related memory changes, as well as neurodegenerative conditions [13]. TOAs must not have scored in the impaired range (*z* –1.5 or below) for same-aged peers on more than one cognitive test at any of the time points assessed. That is, all TOAs who displayed cognitive impairment based on demographically adjusted norms were excluded. The neuropsychological tests used to determine SCP and TOA classification were, Logical Memory [14], Rey Complex Figure Test delay [15], Stroop speed of colors/speed of dots [16], Digit Span [17], Digit Symbol Coding [17], Controlled Oral Word Association Task [16], Category Fluency - animals + names [16], Fruit and Furniture - fluency switching [18], and Boston Naming Test [19]. Normative data for all tests were taken from the respective published manuals unless otherwise indicated by Ellis et al. [10], and Supplementary Table 1 details the mean standardized scores for each group for these tests at baseline.

### Magnetic resonance imaging

Participants underwent T1 weighted MRI using the ADNI 3-dimensional (3D) Magnetization Prepared Rapid Gradient Echo (MPRAGE) sequence on 3T scanners at least once over five time points. MPRage images were acquired on 3 scanners with parameters as follow: Siemens Verio (TE = 2.98 ms, TR = 2300 ms, FA = 9°,resolution = 1×1×1.2 mm), Siemens Tim Trio (TE = 2.98 ms, TR = 2300 ms, FA =9°, resolution = 1×1×1.2 mm), Siemens Avanto (TE = 3.05 ms, TR = 2300 ms, FA = 9°, resolution =1×1×1.2 mm). All T1 weighted images were processed using FreeSurfer software version 5.3.0 [http://surfer.nmr.mgh.harvard.edu/;20]. To improve the reliability of longitudinal measures, the T1 weighted images of each subjects were analyzed using the longitudinal stream of FreeSurfer [21]. There were no manual adjustments required for MRI scans from this cohort, with no scans excluded based on poor signal-to-noise ratio. Excessive motion was evaluated visually, and no subjects were removed due to motion artifacts. The Desikan-Killiany atlas was used to compute the 40 cortical thickness and 47 volume measures. Cortical thickness is only able to be reported where the thickness between grey matter and white matter can be measured, for certain areas there is no valid way to measure thickness, and therefore only volume is reported. Individual participant scans were taken approximately 18 months apart; however, this varied between approximately 15 and 21 months per participant. There was no difference in the time intervals between SCP and TOA participants.

### Positron emission tomography

PET neuroimaging was conducted using one of the following Aβ radiotracers: ^11^C-Pittsburgh compound-B (PiB), ^18^F-Florbetapir (FBP), or ^18^F-Flutemetamol (FLUTE). PET methods and procedures have been reported previously [22, 23]. Briefly, a 20-min acquisition was performed 40 min post-injection of PiB, 50 min post-injection of FBP and 90 min post-injection of FLUTE. Standardized uptake value (SUV) data were summed and normalized to a reference region (the cerebellar cortex for PiB, the whole cerebellum for FBP, and the pons for FLUTE) to generate a SUV ratio (SUVR). CapAIBL was used for SUVR quantification [24]. The accepted cut-off values for significant Aβ deposition vary by radiotracer, for PiB the cut-off utilized was≥1.4, FLUTE≥0.55, and FBP≥1.05. All participants with SUVR above or equal to the cut-offs were classified as Aβ+, and those below the threshold were classified as Aβ–. For the secondary analysis, all participants who had one or more Aβ+ scans were removed from the dataset, with 49 SCPs and 53 TOAs remaining.

### Statistical analysis

Statistical analyses were performed using R version 3.5.1 (R Foundation for Statistical Computing, Vienna, Austria). A *p-*value < 0.05 determined a significant result, and all *p*-values were adjusted for multiple comparisons using false discovery rate (FDR).

Means, standard deviations and percentages are provided for all SCPs and TOAs, as well as for the Aβ- subgroups, with independent sample *t*-tests and chi square (*χ*^2^) analyses as appropriate conducted to evaluate group differences. A series of repeated measures linear mixed effects model (LMM) analyses (using maximum likelihood estimation and an unstructured covariance matrix) were conducted to examine the relationship between SCP and TOA status, and time (actual time between MRI visits in years) with respect to change in ROI cortical thickness and volume. Age, sex, apolipoprotein (*APOE*) *ɛ*4 allele status (the most common genetic risk factor for AD), and MRI scanner (due to multiple scanners being utilized for MRI scans) were entered as confounders, baseline age^*^sex (to account for baseline differences per participant), SCP/TOA status^*^time interactions to investigate changes in ROI over time dependent upon SCP/TOA status, and a random intercept to account for multiple observations per participant. A random slope was not fitted due to limited sample size. This was repeated for the Aβ- only groups. All participants irrespective of their number of scans were used in the LMM to reduce parameter variation, however only those with three or more time points were used in the calculation of the time related changes between SCP/TOA groups.

## RESULTS

Although the TOA group was not selected to be matched to the SCP group, there were no statistically significant differences in the demographics between groups (Table 2). The 76 SCPs comprised 42% males, whilst the 100 TOAs comprised 44% males. Average age did not differ between the two groups (SCPs: 75.58±3.9 versus TOAs: 76.70±4.4). Thirty-six percent of SCPs and 48% of TOAs had less than 13 years of education, and 20% of SCPs and 23% of TOAs had one or more *APOE* *ɛ*4 alleles.

**Table 2:** Descriptive statistics for superior cognitive performers and typical older adults

	SCP	TOA	*p*-values for SCP and TOA differences	Aβ-SCP	Aβ-TOA	*p*-values for Aβ-SCP and TOA differences
	(*n* = 76)	(*n* = 100)		(*n* = 49)	(*n* = 53)	
Age at baseline, y	75.58±3.9	76.70±4.4	0.830	75.33±3.6	75.74±4.1	0.600
Sex, male; n (%)	32 (42)	44 (44)	0.878	20 (41)	21 (40)	0.689
Education≤12 y; n (%)	27 (36)	48 (48)	0.124	19 (39)	22 (42)	0.840
Presence of *APOE* *ɛ*4 allele; n (%)	15 (20)	23 (23)	0.712	5 (10)	7 (13)	0.547
Country of birth, Australia^*^; n (%)	55 (72)	80 (80)	0.235	36 (73)	39 (74)	0.860
Aβ +ve; n (%)	27 (36)	47 (47)	0.124

If not otherwise described, data are presented as mean±standard deviation of the mean. Aβ, amyloid-β; *APOE*, Apolipoprotein *ɛ*4; SCP, superior cognitive performer; TOA, typical older adult; y, years. Characteristics compared using independent samples *t*-test for continuous variables and *χ*^2^ for categorical variables. ^*^Other countries of birth include United Kingdom, Singapore, Netherlands, Egypt, South Africa, Zimbabwe, Malaysia, Sweden, Germany.

We also analyzed the Aβ- only subgroup. With regard to demographics of these subgroups (that is, Aβ- SCPs and TOAs), again there were no statistically significant differences. Of the 49 SCPs in this subsample, 41% were males; of the 53 TOAs, 40% were males. Average age did not differ between the two groups (SCP: 75.33±3.6 versus TOA: 75.74±4.1). Thirty-nine percent of SCPs and 42% of TOAs had less than 13 years of education, and 10% of SCPs and 13% of TOAs had one or more *APOE* *ɛ*4 alleles (Table 2). The total number of SCPs and TOAs with MRI data available at each time point is reported in Table 3. Of the total number of participants, 88 (51 SCP, 37 TOA) had at least two MRI scans, and 31 participants (18 SCP, 13 TOA) had at least three MRI scans.

**Table 3:** The number of participants with MRIs at each time point for the cohort as a whole and for the subset of only Aβ negative participants

	Time point 1	Time point 2	Time point 3	Time point 4	Time point 5
SCP	24	30	21	41^*^	37
TOA	31	31	21	43	32
Aβ –ve SCP	17	22	13	27	22
Aβ –ve TOA	16	17	10	24	18

Aβ, amyloid-β; AIBL, Australian Imaging, Biomarkers, and Lifestyle study; MRI, magnetic resonance imaging; SCP, superior cognitive performer; TOA, typical older adult. ^*^Note: Only a small number of AIBL participants were imaged at baseline due to funding restraints. As more funding became available over the years, a greater number were imaged. Furthermore, the number of participants at time point 3 was lower than time point 4 as it was at time point 4 where AIBL initiated recruitment of enrichment subjects (participants who were recruited from baseline in 2006 were termed “inception”).

**Fig. 2:**
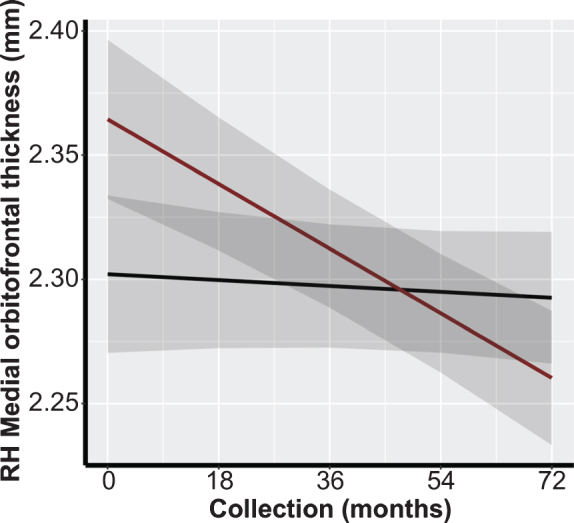
Example representation of differences in cortical thickness trajectory between SCPs and TOAs: right medial orbitofrontal prefrontal cortex FDR, false discovery rate; RH, right hemisphere; SCP, superior cognitive performer, super-ager; TOA, typical older adult. Black Line: SCPs; Red Line: TOAs.

We conducted multiple LMM analyses to assess the association between preservation of brain morphology in SCPs and TOAs in ROIs identified in the previous literature (Table 1). There were no significant differences between both overall cortical thickness and volumetric measures, nor between rates of cortical thickness decline or volume atrophy between SCPs and TOAs in these ROIs preceding or following FDR adjustment (Table 4). In the Aβ- subgroup analysis, preceding FDR adjustment, there was a significant association in the medial orbitofrontal cortical thickness in the right hemisphere (β=–0.0023; *p* = 0.032; Fig. 2). However, following FDR adjustment, results were non-significant (Table 4).

**Table 4:** Results of linear mixed models examining the association between change in cortical thickness and cerebral volume in regions of interest previously identified in published literature and superior cognitive performer status

		Left Hemisphere			Right Hemisphere			Aβ Negative Left Hemisphere			Aβ Negative Right Hemisphere		
Brain area	Volume or Thickness	Beta (SE)	p	FDR Adjusted p	Beta (SE)	p	FDR Adjusted p	Beta (SE)	p	FDR Adjusted p	Beta (SE)	p	FDR Adjusted p
Cerebral Cortex	Thickness	–0.0001 (0.003)	0.979	0.979	–0.0022 (0.003)	0.490	0.920	–0.0022 (0.004)	0.553	0.749	–0.0033 (0.004)	0.389	0.749
Caudal Anterior Cingulate	Thickness	–0.0088 (0.006)	0.175	0.920	–0.0046 (0.006)	0.418	0.920	–0.011 (0.008)	0.149	0.749	–0.0061 (0.007)	0.403	0.749
Inferior Parietal	Thickness				–0.0046 (0.004)	0.294	0.920				–0.0053 (0.005)	0.283	0.749
Isthmus Cingulate	Thickness	–0.0034 (0.006)	0.571	0.920	–0.0090 (0.005)	0.077	0.841	–0.0030 (0.008)	0.697	0.749	–0.0066 (0.006)	0.253	0.749
Lateral Occipital	Thickness				–0.0033 (0.004)	0.348	0.920				–0.0063 (0.004)	0.139	0.749
Lateral Orbitofrontal	Thickness	–0.0058 (0.005)	0.223	0.920	–0.0048 (0.005)	0.366	0.920	–0.0096 (0.006)	0.118	0.749	–0.0028 (0.007)	0.676	0.749
Medial Orbitofrontal	Thickness	0.0023 (0.005)	0.664	0.978	–0.0093 (0.005)	0.087	0.841	–0.0027 (0.007)	0.687	0.749	–0.0153 (0.007)	**0.031**	0.749
Middle Temporal	Thickness				–0.0001 (0.004)	0.972	0.979				–0.0021 (0.005)	0.681	0.749
Pars Opercularis	Thickness	–0.0007 (0.005)	0.877	0.978	–0.0089 (0.005)	0.072	0.841	–0.0064 (0.006)	0.276	0.749	–0.0095 (0.006)	0.108	0.749
Pars Orbitalis	Thickness	–0.0078 (0.006)	0.198	0.920	–0.0039 (0.006)	0.517	0.920	–0.0102 (0.008)	0.178	0.749	–0.0097 (0.007)	0.187	0.749
Pars Triangularis	Thickness	–0.0002 (0.005)	0.972	0.979	–0.0033 (0.005)	0.549	0.920	–0.0030 (0.007)	0.652	0.749	–0.0049 (0.007)	0.490	0.749
Peri Calcarine	Thickness	–0.0015 (0.005)	0.772	0.978	–0.0010 (0.005)	0.853	0.978	–0.0018 (0.006)	0.760	0.787	0.0005 (0.006)	0.934	0.934
Post Central	Thickness				0.0006 (0.004)	0.870	0.978				–0.0034 (0.004)	0.454	0.749
Posterior Cingulate	Thickness	–0.0013 (0.005)	0.799	0.978	–0.0052 (0.004)	0.165	0.920	0.0030 (0.006)	0.617	0.749	–0.0021 (0.005)	0.664	0.749
Rostral Anterior Cingulate	Thickness				–0.0040 (0.006)	0.509	0.920				–0.0045 (0.007)	0.526	0.749
Rostral Middle Frontal	Thickness	–0.0012 (0.004)	0.769	0.978	0.0009 (0.004)	0.833	0.978	–0.0053 (0.005)	0.288	0.749	–0.0026 (0.006)	0.640	0.749
Superior Frontal	Thickness	–0.0025 (0.004)	0.571	0.920	–0.0027 (0.004)	0.518	0.920	–0.0035 (0.005)	0.472	0.749	–0.0029 (0.005)	0.570	0.749
Insula	Thickness	–0.0041 (0.004)	0.333	0.920	0.0004 (0.005)	0.946	0.978	–0.0030 (0.005)	0.565	0.723	–0.0028 (0.007)	0.683	0.723
Cerebral Cortex	Volume	0.0075 (0.024)	0.752	0.978	–0.0087 (0.024)	0.721	0.978	–0.0054 (0.030)	0.857	0.787	–0.0181 (0.031)	0.559	0.723
Hippocampus	Volume	–0.0005 (< 0.001)	0.301	0.920	–0.0003 (< 0.001)	0.489	0.920	–0.0004 (0.001)	0.428	0.749	–0.0006 (0.001)	0.367	0.723

Beta coefficients represent the comparison of brain region cortical thickness and volume over time between typical older adult and superior cognitive performers with associated standard error Aβ, amyloid-β; FDR, false discovery rate; SE, standard error. **Bold** indicates statistical significance at *p* < 0.05 before FDR Adjustment. A negative β coefficient indicates volume/cortical atrophy over time, with strong negative coefficients indicating greater atrophy relative to the error and sample size.

Following the initial analyses, additional regions not previously identified as important in the SCP literature were examined (45 volume and 22 cortical thickness regions). Assessing both the main effects for differences in cortical thickness and volumetric measures, and the atrophy in either cortical thickness or volumetric measures over time, we found only one region, right hemisphere rostral anterior cingulate, with significantly greater volume (β= –0.0142, *p* > 0.001) in SCPs as compared with TOAs. While there was an overall difference in volume between groups, the rate of change in volume in this region was not significantly different over time (*p* > 0.050). Supplementary Table 4 provides the main effects results from both the ROI and additional analyses in the cohort as a whole. At the nominal significance level, there were two associations observed between rate of volume atrophy between SCPs and TOAs in the right banks of the superior temporal sulcus (β= –0.0007; *p* = 0.025) and left hemisphere lateral orbitofrontal cortex volume (β  = 0.0033; *p* = 0.021). However, following FDR adjustment, no significant differences in these regions remained (Supplementary Tables 2–4).

In the Aβ- subgroup analysis, higher volume loss was observed in the TOAs compared with the SCPs in the right banks of the superior temporal sulcus (β= –0.0011; *p* = 0.006) and the right hemisphere medial orbitofrontal region (β= –0.0023; *p* = 0.032). However following FDR adjustment, these differences were no longer significant (Supplementary Tables 2 and 3).

Given the sample size of the two separate groups with three or more MRI measures, and the longitudinal nature of the study, we computed an estimated required sample size for the LMM to be able to detect a significant difference in cortical thickness atrophy over time [25]. We used one region for volume (left hemisphere cortical volume) and one region for cortical thickness (right hemisphere cortical thickness) and using the power calculations adapted from Liu & Liang et al. (1997) computed the sample size (using alpha at 0.05, and 80% power) that would be needed to be able to find a difference between the two slopes representing cortical thickness atrophy. Rate of cortical volume loss per year for a healthy normal population over the age of 60 has been estimated to be approximately 0.5% per year [26, 27]. If a SCP participant lost up to ∼50% less volume than a typical ager (0.25% /year), then using this approximated attenuation of volume loss, and the model estimates given by the LMM (up to three time points, adjusting for age, gender, *APOE* *ɛ*4 allele status and scanner), the required sample size to detect a difference between the two slopes (SCP versus TOA) would be > 8,000 participants per group. Changing the magnitude of the difference in atrophy either up or down around 50% either decreases or increases the expected sample size respectively. Such large expected sample sizes demonstrate that the proportion of variance in the MRI measure to the small changes in volume which may occur between the SCP/TOA group is too large to see any significant changes over time.

As described above, Rogalski’s [5] definition of SA utilizes those aged 80 + at baseline, and potentially the SA phenomenon may only be expressed after this age. In the current study, the sample of participants 80 + was too small to attain meaningful statistical results using longitudinal analysis, but a cross-sectional between-groups analysis of a subsample of this cohort was completed using the first MRI scan obtained after reaching 80. There were 20 participants in the SCP group, and 26 participants in the TOA group of the present cohort who turned 80 years old during the study time frame and were therefore included in this additional analysis. Of interest was the relatively high proportion of the SCP group who were Aβ+(40%) as compared with the TOA group (69%), although this did not reach statistical significance (*χ*^2^ = 2.83, *p* = 0.092). MRI scan results for previously identified neural regions did not differ significantly for volume or thickness between groups in models adjusting for age, gender, and *APOE* *ɛ*4 allele status (data not shown).

## DISCUSSION

This study provides several novel contributions to the literature examining older adults with superior cognitive performance. Specifically, to the authors’ knowledge, this is the first published study to evaluate brain morphology in SCPs compared to TOAs, 1) longitudinally, with a relatively large sample size over an extended period (76 SCPs and 100 TOAs followed for up to 72 months) with detailed specific neural regions, 2) ensuring reliable classification of SCP and TOA by requiring stability of cognitive performance over the course of the study and excluding individuals displaying non-age-related cognitive decline/emergent AD, and 3) extending the literature on SCPs by evaluating the role of Aβ load.

In summary, we evaluated longitudinal trajectories of cortical thickness and volume by comparing preservation of ROIs identified as potentially relevant to SCP status in the extant literature, as well as discovery-phase analyses of additional neural regions. After adjusting for FDR, there were no significant differences in the rate of cortical thickness decline or volume atrophy between SCPs and TOAs in any neural regions.

Our results were partially consistent with Harrison et al. [6] who found that the longitudinal rate of hippocampal volume atrophy and whole cortex cortical thinning was not significantly different between successful agers and TOAs. Of note, however, Harrison et al. [6] also concluded that their successful agers displayed attenuated left hemisphere cortical thinning in the anterior cingulate cortex, middle cingulate, medial prefrontal, and insula over an average follow up period of 4.5±2.7 years for successful agers and 3.5±1.9 years for TOAs. Additionally, our findings were not consistent with the small number of cross-sectional studies of older adults with SCP (e.g., [1–3], which appeared to show that there were significant differences in cortical thickness and/or volume between those with SCP and TOAs in multiple neural regions. Of note, this was true both in the primary longitudinal analyses using an age cut-off of 70+, as well as in the *post-hoc* cross-sectional analysis evaluating only those participants 80 + .

While our sample sizes were generally larger than in most previous SCP studies, our statistical power to detect small effect sizes was still relatively limited. Therefore, we have also presented initial results unadjusted for multiple comparisons. While these exploratory observations must be interpreted with caution, they may be helpful in guiding and/or interpreting future studies of super-aging with larger sample sizes. Specifically, results prior to FDR adjustment suggested that SCPs had decreased cerebral volume atrophy in the right banks of the superior temporal sulcus (STS) and left lateral orbitofrontal cortex. Of note, these associations were observed in the discovery phase and these findings have not been observed in previous published research. Implications of these observations will be described below.

Previous studies of SCP have been limited by the lack of inclusion of amyloid imaging to allow for exclusion of individuals in both the SCP and TOA groups with potential preclinical AD. A novel contribution of the present study was the longitudinal sub-analyses with only cerebral Aβ- participants, thereby removing those individuals with increased risk of developing AD. In the ROI phase of the analyses and preceding FDR adjustment, attenuated cortical thinning was observed in the right hemisphere medial orbitofrontal region in the SCPs. In addition, in the discovery phase analyses in SCPs, cerebral volume preservation was seen in the right hemisphere medial orbitofrontal region for Aβ- participants. Although not statistically significant, these finding are similar to those reported by Sun et al. [3] and Harrison et al. [6], who found significantly decreased cortical thickness in TOAs compared to SAs/successful agers in the medial prefrontal cortex more broadly. Finally, our exploratory analyses revealed cerebral volume preservation in the right banks of the STS, which had not been observed in previous studies of super-aging. Of note, however, while the evaluation of an entirely amyloid negative group of SCPs was a strength, the sample size of this distinct group remained quite limited and the potential role of amyloid status in this construct needs further investigation.

The regions identified in the current study as possible regions of interest (the orbitofrontal cortices and right STS) are notable for mediating neural processes that may be relevant for super-aging, including superior memory and executive functions. For example, Wilson and colleagues state that the anterior dorsal bank of the STS contains representations reflecting patterns of activity that “encode abstract lexical entries, but do not contain phonological, orthographic, semantic or syntactic information. Rather, these lexical nodes serve as hubs that bind together these different types of information” [27]. Insights into the importance of these areas to the maintenance of superior memory can be inferred from studies which show damage to this region interferes with the links between meanings and word forms, but not with the meanings and word forms themselves [28]. Therefore, this region appears to contribute vital neural connections between words and their conceptual underpinnings, and volume loss may be associated with difficulty in retrieval or recognition of verbal information due to the interruption of these linkages.

Bilateral medial and lateral orbitofrontal cortex thickness and volume were also identified as potentially associated with superior cognitive performance status in the present study. These regions are involved in adaptive and goal-directed behavior, including decision-making [29]. In particular, the medial orbitofrontal cortex is thought to be important in regulating goal-directed response selection, which involves predicting the consequences of one’s actions and the value of potential payoffs [30]. The medial prefrontal region, of which the orbitofrontal cortex lies within, is part of the default mode circuitry, a major intrinsic brain network. The default mode network is implicated in episodic memory processes including encoding, storage, and retrieval [3].

Age-related atrophy is particularly prominent in a number of regions including the prefrontal cortex [31, 32], and it is conceivable that loss of volume and/or cortical thickness in this region would result in a decline in episodic memory due to the integrity of the default mode network being compromised. Of note, however, these conclusions must be interpreted with caution due to the lack of significant findings post FDR adjustment, perhaps due to our limited sample size.

Another novel contribution of the present study is the methodological rigor used for group classification. This is the first study that identified SCPs and TOAs based on comprehensive and longitudinal stability of cognitive performance and functional ability. This likely increased the reliability of the SCP classification and removed individuals from the TOA group with preclinical dementia status. We believe that this is an important step forward in the study of the SCP construct, which increases the confidence in our current pattern of results. Previous cross-sectional studies have not been able to confidently exclude “at risk” individuals from their TOA group, and it is quite likely that without amyloid imaging or longitudinal data a subset of their TOA group would include such individuals. Further our *post-hoc* cross-sectional analysis of participants 80+, produced similar results to the primary analysis. The finding that 40% of the aged 80 + SCP group are Aβ+ is of interest, as these participants have amyloid pathology; however, still perform exceptionally well at multiple timepoints. Such a cohort will be worth following to define predictors of resilience to amyloid pathology in a larger sample size.

Our findings suggest that even older adults (aged 70+) who maintain a high level of memory function and perform at a level 25 or more years younger than their peer group, still show age-related changes in brain morphology similar to peers with age-consistent memory functions. Therefore, the question remains as to how these individuals maintain this level of cognitive function whilst brain changes are progressing at a normal rate. Cognitive reserve (CR) is a likely factor in accounting for the findings in the extant SCP literature.

Cognitive reserve is developed by a lifetime of education and cognitively enriching experiences to help the brain better cope with any failures or declines it faces. Cognitive reserve provides resilience to acquired neuropathology, and therefore cognitive and functional changes develop at a later stage for individuals with high CR compared to individuals with low CR despite similar levels of neuropathology [33]. This is to be contrasted with one view of the super-aging construct, in which SAs are thought to be resilient to the development of neuropathology itself well into advanced age. For example, an autopsy study of SAs showed decreased AD pathological changes compared to TOAs, and the authors concluded that a decrease in AD pathology may underlie SAs resistance to changes in memory performance [34]. However, Harrison et al. [6] acknowledge that it remains unclear whether successful agers avoid neuropathology or are simply resilient to the effects of such pathologies for an undetermined length of time, and that this remains an under-explored area of research. It could be argued that the findings of the present study support the latter explanation—that is, that successful agers remain resilient to the cognitive and behavioral consequences of neuropathology for an extended period of time. That is, they display high CR. Of note, the SCPs and TOAs in the current study did not differ in level of education (percentage of those with less than or equal to 12 years of education, and percentage with greater than 12 years of education), and education level is often used as a proxy for cognitive reserve. However, education is generally considered a crude and imperfect indicator of CR, and some have argued for alternative indicators (e.g., [35]). It will be important for future research to more comprehensively assess potential differences in cognitive reserve between those with SCP and TOAs.

Of note, however, given that the present results from LMMs defined the rate of atrophy for both TOA and SCP participants to be very similar, *post-hoc* sample size calculations revealed that to find a clinically meaningful difference, studies would need an unrealistic number of participants to be followed up over a similar time period. In summary, despite the lack of statistically significant brain morphological differences between SCPs and TOAs reported in the present study, our findings do not preclude the possibility of super-agers, as contrasted from a group of individuals with high brain or cognitive reserve.

The present study also expanded on our understanding of the way amyloid load fits into the SCP construct initiated in Dang et al. [8]. We observed the SCPs and TOAs did not significantly differ on percentage of those classed as Aβ+, and the results did not differ when the Aβ- sub sample was analyzed independently.

There are limitations to our findings; specifically, a relatively small sample size of 76 SCPs and 100 TOAs were studied, with a maximum of 41 and 43 participants in each group with MRI data at any one time point, reducing further when 3 or more MRI scan were included. However, *post-hoc* sample size calculations indicate that very large samples would be needed to be able to detect a clinically meaningful and significant difference in the atrophy slopes between these two groups. This sample size problem remains a challenge in any study of super-aging, which is thought to be relatively rare, even in large cohort studies such as AIBL. When individuals that are Aβ+ are excluded, the numbers decrease even further. Very large cohort studies will be required to more thoroughly evaluate the validity of the super-aging construct.

In summary, we here reported that SCPs and TOAs in our cohort did not display differing trajectories of cortical thinning and volume atrophy over six years, even when only Aβ- participants were analyzed. The findings from this cohort provide preliminary evidence that whilst maintenance of superior memory may be a biological possibility, it may not coincide with decreased cerebral changes associated with aging, including cortical thinning and volume atrophy. Further longitudinal studies with amyloid negative participants are required to replicate our findings. Future research will additionally elucidate potential pathways for the resistance of SCPs to age-related cognitive, identify factors that promote successful aging, and evaluate whether these relationships are consistent across racial, ethnic, and socioeconomic groups.

## Supplementary Material

Supplementary Material

## References

[1] Harrison TM , Weintraub S , Mesulam MM , Rogalski E (2012) Superior memory and higher cortical volumes in unusually successful cognitive aging. J Int Neuropsychol Soc 18, 1081–1085. 23158231 10.1017/S1355617712000847 PMC3547607

[2] Fjell AM , Walhovd KB , Reinvang I , Lundervold A , Salat D , Quinn BT , Fischl B , Dale AM (2006) Selective increase of cortical thickness in high-performing elderly–structural indices of optimal cognitive aging.. Neuroimage 29, 984–994. 16176876 10.1016/j.neuroimage.2005.08.007

[3] Sun FW , Stepanovic MR , Andreano J , Barrett LF , Touroutoglou A , Dickerson BC (2016) Youthful brains in older adults: Preserved neuroanatomy in the default mode and salience networks contributes to youthful memory in superaging. J Neurosci 36, 9659–9668. 27629716 10.1523/JNEUROSCI.1492-16.2016 PMC5039247

[4] Gefen T , Peterson M , Papastefan ST , Martersteck A , Whitney K , Rademaker A , Bigio EH , Weintraub S , Rogalski E , Mesulam MM , Geula C (2015) Morphometric and histologic substrates of cingulate integrity in elders with exceptional memory capacity. J Neurosci 35, 1781–1791. 25632151 10.1523/JNEUROSCI.2998-14.2015 PMC4308613

[5] Rogalski E , Gefen T , Mao Q , Connelly M , Weintraub S , Geula C , Bigio EH , Mesulam MM (2019) Cognitive trajectories and spectrum of neuropathology in SuperAgers: The first 10 cases. Hippocampus 29, 458–467. 29341318 10.1002/hipo.22828 PMC6050141

[6] Harrison TM , Maass A , Baker SL , Jagust WJ (2018) Brain morphology, cognition, and beta-amyloid in older adults with superior memory performance. Neurobiol Aging 67, 162–170. 29665578 10.1016/j.neurobiolaging.2018.03.024 PMC5955827

[7] Cook AH , Sridhar J , Ohm D , Rademaker A , Mesulam MM , Weintraub S , Rogalski E (2017) Rates of cortical atrophy in adults 80 years and older with superior vs average episodic memory. JAMA 317, 1373–1375. 28384819 10.1001/jama.2017.0627 PMC5847263

[8] Dang C , Yassi N , Harrington KD , Xia Y , Lim YY , Ames D , Laws SM , Hickey M , Rainey-Smith S , Sohrabi HR , Doecke JD , Fripp J , Salvado O , Snyder PJ , Weinborn M , Villemagne VL , Rowe CC , Masters CL , Maruff P , AIBL Research Group (2019) Rates of age- and amyloid beta-associated cortical atrophy in older adults with superior memory performance. Alzheimers Dement (Amst) 11, 566–575. 31909172 10.1016/j.dadm.2019.05.005 PMC6939054

[9] Salat DH , Buckner RL , Snyder AZ , Greve DN , Desikan RS , Busa E , Morris JC , Dale AM , Fischl B (2004) Thinning of the cerebral cortex in aging. Cereb Cortex 14, 721–730. 15054051 10.1093/cercor/bhh032

[10] Ellis KA , Bush AI , Darby D , De Fazio D , Foster J , Hudson P , Lautenschlager NT , Lenzo N , Martins RN , Maruff P , Masters C , Milner A , Pike K , Rowe C , Savage G , Szoeke C , Taddei K , Villemagne V , Woodward M , Ames D (2009) The Australian Imaging, Biomarkers and Lifestyle (AIBL) study of aging: Methodology and baseline characteristics of 1112 individuals recruited for a longitudinal study of Alzheimer’s disease. Int Psychogeriatr 21, 672–687. 19470201 10.1017/S1041610209009405

[11] Rogalski E (2019) Don’t forget—Age is a relevant variable in defining SuperAgers.. Alzheimers Dement (Amst) 11, 560–561. 31909169 10.1016/j.dadm.2019.05.008 PMC6939045

[12] Pike KE , Ellis KA , Villemagne VL , Good N , Chetelat G , Ames D , Szoeke C , Laws SM , Verdile G , Martins RN , Masters CL , Rowe CC (2011) Cognition and beta-amyloid in preclinical Alzheimer’s disease: Data from the AIBL study. Neuropsychologia 49, 2384–2390. 21529702 10.1016/j.neuropsychologia.2011.04.012

[13] Rowe CC , Bourgeat P , Ellis KA , Brown B , Lim YY , Mulligan R , Jones G , Maruff P , Woodward M , Price R , Robins P , Tochon-Danguy H , O’Keefe G , Pike KE , Yates P , Szoeke C , Salvado O , Macaulay SL , O’Meara T , Head R , Cobiac L , Savage G , Martins R , Masters CL , Ames D , Villemagne VL (2013) Predicting Alzheimer disease with beta-amyloid imaging: Results from the Australian imaging, biomarkers, and lifestyle study of ageing. Ann Neurol 74, 905–913. 24448836 10.1002/ana.24040

[14] Wechsler D (1945) A standardised memory scale for clinical use. J Psychol 19, 87–95.

[15] Meyers JE , Meyers KR (1995) *Rey Complex Figure Test and Recognition Trial. Professional Manual*, Psychological Assessment Resource, Inc.

[16] Strauss E , Sherman , Spreen O (2006) A Compendium of Neuropsychological Tests: Administration, Norms, and Commentary (3rd edn), Oxford University Press, New York.

[17] Wechsler D (1997) Wechsler Adult Intelligence Scale, 3rd edition (WAIS-III), Psychological Corporation, San Antonio, TX.

[18] Delis DC , Kaplan E , Kramer JH (2001) Delis-Kaplan Executive Funtion System (D-KEFS), Psychological Corporation, San Antonio, TX.

[19] Saxton J , Ratcliff G , Munro CA , Coffey EC , Becker JT , Fried L , Kuller L (2000) Normative data on the Boston Naming Test and two equivalent 30-item short forms. Clin Neuropsychol 14, 526–534. 11262721 10.1076/clin.14.4.526.7204

[20] Fischl B (2012) FreeSurfer. Neuroimage 62, 774–781. 22248573 10.1016/j.neuroimage.2012.01.021 PMC3685476

[21] Reuter M , Schmansky NJ , Rosas HD , Fischl B (2012) Within-subject template estimation for unbiased longitudinal image analysis. Neuroimage 61, 1402–1418. 22430496 10.1016/j.neuroimage.2012.02.084 PMC3389460

[22] Rowe CC , Ellis KA , Rimajova M , Bourgeat P , Pike KE , Jones G , Fripp J , Tochon-Danguy H , Morandeau L , O’Keefe G , Price R , Raniga P , Robins P , Acosta O , Lenzo N , Szoeke C , Salvado O , Head R , Martins R , Masters CL , Ames D , Villemagne VL (2010) Amyloid imaging results from the Australian Imaging, Biomarkers and Lifestyle (AIBL) study of aging. Neurobiol Aging 31, 1275–1283. 20472326 10.1016/j.neurobiolaging.2010.04.007

[23] Villemagne VL , Doré V , Yates P , Brown B , Mulligan R , Bourgeat P , Veljanoski R , Rainey-Smith SR , Ong K , Rembach A , Williams R , Burnham SC , Laws S , Salvado O , Taddei K , Macaulay SL , Martins RN , Ames D , Masters CL , Rowe CC (2014) En attendant centiloid. Adv Res 2, 723–729.

[24] Bourgeat P , Villemagne VL , Dore V , Brown B , Macaulay SL , Martins R , Masters CL , Ames D , Ellis K , Rowe CC , Salvado O , Fripp J , AIBL Research Group (2015) Comparison of MR-less PiB SUVR quantification methods. Neurobiol Aging 36, S159–166. 25257985 10.1016/j.neurobiolaging.2014.04.033

[25] Donohue MC (2019) longpower: Power and sample size calculations for longitudinal data. R package version 1.0-19.

[26] Fjell AM , Walhovd KB , Fennema-Notestine C , McEvoy LK , Hagler DJ , Holland D , Brewer JB , Dale AM (2009) One-year brain atrophy evident in healthy aging. J Neurosci 29, 15223–15231. 19955375 10.1523/JNEUROSCI.3252-09.2009 PMC2827793

[27] Wilson SM , Bautista A , McCarron A (2018) Convergence of spoken and written language processing in the superior temporal sulcus. Neuroimage 171, 62–74. 29277646 10.1016/j.neuroimage.2017.12.068 PMC5857434

[28] Schwartz MF , Kimberg DY , Walker GM , Faseyitan O , Brecher A , Dell GS , Coslett HB (2009) Anterior temporal involvement in semantic word retrieval: Voxel-based lesion-symptom mapping evidence from aphasia. Brain 132, 3411–3427. 19942676 10.1093/brain/awp284 PMC2792374

[29] Nogueira R , Abolafia JM , Drugowitsch J , Balaguer-Ballester E , Sanchez-Vives MV , Moreno-Bote R (2017) Lateral orbitofrontal cortex anticipates choices and integrates prior with current information. Nat Commun 8, 14823. 28337990 10.1038/ncomms14823 PMC5376669

[30] Gourley SL , Zimmermann KS , Allen AG , Taylor JR (2016) The medial orbitofrontal cortex regulates sensitivity to outcome value. J Neurosci 36, 4600–4613. 27098701 10.1523/JNEUROSCI.4253-15.2016 PMC4837686

[31] McGinnis SM , Brickhouse M , Pascual B , Dickerson BC (2011) Age-related changes in the thickness of cortical zones in humans. Brain Topogr 24, 279–291. 21842406 10.1007/s10548-011-0198-6 PMC3600370

[32] Bakkour A , Morris JC , Wolk DA , Dickerson BC (2013) The effects of aging and Alzheimer’s disease on cerebral cortical anatomy: Specificity and differential relationships with cognition. Neuroimage 76, 332–344. 23507382 10.1016/j.neuroimage.2013.02.059 PMC4098706

[33] Stern Y (2002) What is cognitive reserve? Theory and research application of the reserve concept. J Int Neuropsychol Soc 8, 448–460. 11939702

[34] Rogalski EJ , Gefen T , Shi J , Samimi M , Bigio E , Weintraub S , Geula C , Mesulam MM (2013) Youthful memory capacity in old brains: Anatomic and genetic clues from the Northwestern SuperAging Project. J Cogn Neurosci 25, 29–36. 23198888 10.1162/jocn_a_00300 PMC3541673

[35] Reed BR , Mungas D , Farias ST , Harvey D , Beckett L , Widaman K , Hinton L , DeCarli C (2010) Measuring cognitive reserve based on the decomposition of episodic memory variance. Brain 133, 2196–2209. 20591858 10.1093/brain/awq154 PMC3139935

