# Supplementary material for: Longitudinal Trajectories in Cortical Thickness and Volume Atrophy: Superior Cognitive Performance Does Not Protect Against Brain Atrophy in Older Adults
Source: J Alzheimers Dis. 2021 Jun 1;81(3):1039–52. doi: 10.3233/JAD-201243 (PMC8293653; doi:10.3233/JAD-201243)
Supplement: Supplementary Material [file jad-81-jad201243-s001.pdf]

Supplementary Material

Longitudinal Trajectories in Cortical Thickness and Volume Atrophy: Superior Cognitive Performance Does Not Protect Against Brain Atrophy in Older Adults

Supplementary Table 1. Cognitive normative test *z* and scaled scores for superior cognitive performers and typical older adults at baseline.

	SCP (n = 76)	TOA (n = 100)	p for SCP and TOA differences
**CVLT Delayed Recall**	1.58 ± 0.6	0.52 ± 0.8	<0.000
**RCFT 30-min Delay**	1.21 ± 1.6	0.54 ± 1.3	0.002
**Digit Span***	13.00 ± 3.1	12.01 ± 2.8	0.031
**Digit Symbol Coding***	12.77 ± 2.7	11.60 ± 2.5	0.004
**Controlled Oral Word Association Task***	13.39 ± 3.0	11.86 ± 3.4	0.002
**Category Fluency – Animals + Names***	13.60 ± 3.0	12.47 ± 2.9	0.015
**Switching Fruit/Furniture***	12,79 ± 2.6	11.78 ± 2.7	0.013
**Boston Naming Test**	0.89 ± 0.6	0.77 ± 0.5	0.191
**STROOP C/D**	- 0.41 ± 0.7	-0.36 ± 0.8	0.656

Data are presented as mean ± standard deviation of the mean.
CVLT, California Verbal Learning Test; RCFT, Rey Complex Figure Test and Recognition Trial; SCP, superior cognitive performer; TOA, typical older adult.
Characteristics compared using independent samples t-test.
* Age scaled scores (mean = 10, standard deviation = 3)

Supplementary Table 2. Results of linear mixed models examining the association between change in cortical thickness in regions of interest not previously identified in published literature on superior cognitive performance.

	Left Hemisphere			Right Hemisphere			Aβ -ve Left Hemisphere			Aβ -ve Right Hemisphere		
**Brain area**	**Beta (SE)**	**p**	**FDR Adjusted p**	**Beta (SE)**	**p**	**FDR Adjusted p**	**Beta (SE)**	**p**	**FDR Adjusted p**	**Beta (SE)**	**p**	**FDR Adjusted p**
**Caudal Middle Frontal**	-0.0013 (0.005)	0.784	0.996	-0.0024 (0.006)	0.659	0.996	-0.0057 (0.006)	0.339	0.994	-0.0017 (0.007)	0.801	0.994
**Cuneus**	-0.0018 (0.004)	0.658	0.996	-0.0001 (0.004)	0.978	0.996	-0.0026 (0.005)	0.601	0.994	-0.0018 (0.005)	0.721	0.994
**Entorhinal**	0.0078 (0.009)	0.383	0.996	-0.0018 (0.008)	0.816	0.996	0.0081 (0.011)	0.481	0.994	0.0010 (0.009)	0.916	0.994
**Fusiform**	-0.0008 (0.004	0.861	0.996	-0.0043 (0.005)	0.359	0.996	-0.0042 (0.005)	0.447	0.994	-0.0083 (0.006)	0.148	0.994
**Inferior Parietal**	0.0009 (0.004)	0.824	0.996				-0.0043 (0.005)	0.384	0.994			
**Inferior Temporal**	-0.0015 (0.004)	0.714	0.996	0.0019 (0.004)	0.666	0.996	0.0018 (0.005)	0.740	0.994	0.0001 (0.005)	0.992	0.994
**Lateral Occipital**	0.0078 (0.004)	0.055	0.996				0.0063 (0.005)	0.210	0.994			
**Lingual**	-0.0012 (0.003)	0.736	0.996	-0.0011 (0.003)	0.756	0.996	-0.0004 (0.004)	0.932	0.994	-0.0022 (0.004)	0.597	0.994
**Middle Temporal**				0.0004 (0.004)	0.926	0.996				0.0013 (0.005)	0.802	0.994
**Parahippocampal**	0.0004 (0.006)	0.948	0.996	-0.0061 (0.005)	0.259	0.996	0.0013 (0.007)	0.853	0.994	-0.0045 (0.007)	0.502	0.994
**Paracentral**	-0.0006 (0.006)	0.929	0.996	0.0068 (0.006)	0.255	0.996	-0.0007 (0.008)	0.912	0.994	0.0107 (0.008)	0.174	0.994
**Post Central**				-0.0012 (0.005)	0.805	0.996				-0.0018 (0.006)	0.758	0.994
**Precentral**	0.0071 (0.006)	0.271	0.996	-0.0025 (0.006)	0.702	0.996	0.0059 (0.008)	0.454	0.994	-0.0007 (0.008)	0.930	0.994
**Precuneus**	-0.0012 (0.004)	0.787	0.996	-0.0026 (0.004)	0.512	0.996	-0.0051 (0.005)	0.281	0.994	-0.007 (0.005)	0.148	0.994
**Rostral Anterior Cingulate**	-0.0061 (0.006)	0.338	0.996				-0.0074 (0.008)	0.380	0.994			
**Superior Parietal**	0.002 (0.0044)	0.651	0.996	-0.0005 (0.004)	0.900	0.996	-0.0023 (0.005)	0.612	0.994	-0.0047 (0.005)	0.320	0.994
**Superior Temporal**	-0.0019 (0.004)	0.624	0.996	-0.0017 (0.004)	0.682	0.996	-0.0016 (0.005)	0.752	0.994	-0.0014 (0.005)	0.794	0.994
**Supramarginal**	0.0001 (0.004)	0.980	0.996	-0.0019 (0.005)	0.706	0.996	-0.0024 (0.005)	0.624	0.994	-0.0015 (0.006)	0.795	0.994
**Frontal Pole**	< 0.001 (0.009)	0.996	0.996	-0.0132 (0.009)	0.147	0.996	0.0066 (0.011)	0.561	0.994	-0.0135 (0.012)	0.246	0.994
**Temporal Pole**	0.0007 (0.009)	0.938	0.996	0.0051 (0.008)	0.526	0.996	0.0043 (0.010)	0.676	0.994	0.0054 (0.010)	0.588	0.994
**Transverse Temporal**	0.0011 (0.007)	0.884	0.996	-0.0143 (0.009)	0.117	0.996	-0.0028 (0.009)	0.758	0.994	-0.0183 (0.012)	0.123	0.994
**Banks of the**	-0.0034	0.464	0.996	-0.0074	0.164	0.996	-0.0057	0.358	0.994	-0.0087	0.166	0.994

**Superior Temporal Sulcus**	(0.005)	(0.005)	(0.006)	(0.006)

Aβ, amyloid-β; FDR, false discovery rate; SE, standard error. A negative β coefficient indicates volume/cortical atrophy over time, with strong negative coefficients indicating greater atrophy relative to the error and sample size.

Supplementary Table 3. Results of linear mixed models examining the association between change in cerebral volume in regions of interest not previously identified in published literature on superior cognitive performance.

	Left Hemisphere			Right Hemisphere			Aβ -ve Left Hemisphere			Aβ -ve Right Hemisphere		
**Brain area**	**Beta (SE)**	**p**	**FDR Adjusted p**	**Beta (SE)**	**p**	**FDR Adjusted p**	**Beta (SE)**	**p**	**FDR Adjusted p**	**Beta (SE)**	**p**	**FDR Adjusted p**
**Caudal Anterior Cingulate**	-0.0001 (<0.001)	0.815	0.996	-0.0002 (<0.001)	0.628	0.996	-0.0004 (<0.001)	0.248	.994	-0.0003 (<0.001)	0.461	0.994
**Caudal Middle Frontal**	<0.0001 (0.001)	0.987	0.996	-0.0005 (0.001)	0.634	0.996	-0.0008 (0.001)	0.518	0.994	-0.0003 (0.001)	0.787	0.994
**Cuneus**	0.0003 (<0.001)	0.510	0.996	0.0001 (<0.001)	0.819	0.996	<0.0001 (0.001)	0.937	0.994	-0.0005 (0.001)	0.390	0.994
**Entorhinal**	0.0003 (0.001)	0.640	0.996	-0.0006 (<0.001)	0.133	0.996	-0.0003 (0.001)	0.636	0.994	-0.0006 (0.001)	0.282	0.994
**Fusiform**	0.0001 (0.001)	0.933	0.996	-0.0013 (0.001)	0.304	0.996	-0.0009 (0.001)	0.524	0.994	-0.0026 (0.001)	0.072	0.994
**Inferior Parietal**	0.0009 (0.002)	0.575	0.996	-0.0015 (0.002)	0.392	0.996	-0.0007 (0.002)	0.702	0.994	-0.0022 (0.002)	0.295	0.994
**Inferior Temporal**	-0.0005 (0.001)	0.692	0.996	-0.0005 (0.001)	0.696	0.996	0.0004 (0.002)	0.782	0.994	-0.0015 (0.002)	0.312	0.994
**Isthmus Cingulate**	0.0005 (<0.001)	0.164	0.996	-0.0005 (<0.001)	0.083	0.996	0.0001 (<0.001)	0.780	0.994	-0.0006 (<0.001)	0.097	0.994
**Lateral Occipital**	0.0033 (0.001)	**0.021**	0.911	-0.0003 (0.001)	0.837	0.996	0.0029 (0.002)	0.102	0.994	-0.0022 (0.002)	0.180	0.994
**Lateral Orbitofrontal**	-0.0010 (0.001)	0.303	0.996	-0.0010 (0.001)	0.345	0.996	-0.0019 (0.001)	0.151	0.994	-0.0012 (0.001)	0.420	0.994
**Lingual**	0.0006 (0.001)	0.432	0.996	0.0006 (0.001)	0.481	0.996	<0.0001 (0.001)	0.967	0.994	-0.0006 (0.001)	0.542	0.994
**Medial Orbitofrontal**	-0.0003 (0.001)	0.778	0.996	-0.0012 (0.001)	0.150	0.996	-0.0015 (0.001)	0.262	0.994	-0.0023 (0.001)	**0.032**	0.994
**Middle Temporal**	-0.0005 (0.001)	0.691	0.996	<0.001 (0.001)	0.982	0.996	-0.0005 (0.001)	0.723	0.994	-0.0002 (0.002)	0.883	0.994
**Parahippocampal**	0.0003 (<0.001)	0.394	0.996	-0.0002 (<0.001)	0.403	0.996	0.0002 (<0.001)	0.552	0.994	-0.0002 (<0.001)	0.467	0.994
**Paracentral**	<0.001 (0.001)	0.941	0.996	0.0008 (0.001)	0.174	0.996	-0.0001 (0.001)	0.831	0.994	0.0009 (0.001)	0.269	0.994
**Pars Opercularis**	0.00017 (0.001)	0.815	0.996	-0.0007 (0.001)	0.233	0.996	-0.0006 (0.001)	0.541	0.994	-0.0003 (<0.001)	0.403	0.994
**Pars Orbitalis**	-0.0002 (<0.001)	0.559	0.996	<0.0001 (<0.001)	0.919	0.996	-0.0004 (<0.001)	0.449	0.994	-0.0003 (0.001)	0.652	0.994
**Pars Triangularis**	<0.0001 (0.001)	0.958	0.996	-0.0001 (0.001)	0.850	0.996	<0.0001 (0.001)	0.994	0.994	-<0.0001 (0.001)	0.982	0.994
**Peri Calcarine**	0.0006 (<0.001)	0.235	0.996	0.0004 (0.001)	0.412	0.996	0.0004 (0.001)	0.404	0.994	0.0004 (0.001)	0.470	0.994

**Post Central**	0.0008 (0.001)	0.549	0.996	-0.0001 (0.001)	0.962	0.996	-0.0003 (0.002)	0.864	0.994	-<0.0001 (0.002)	0.990	0.994
**Posterior Cingulate**	0.0004 (<0.001)	0.290	0.996	-0.0001 (<0.001)	0.889	0.996	0.0003 (<0.001)	0.467	0.994	<0.0001 (<0.001)	0.939	0.994
**Precentral**	0.0016 (0.002)	0.483	0.996	-0.0011 (0.002)	0.622	0.996	0.0010 (0.003)	0.738	0.994	-0.0004 (0.003)	0.885	0.994
**Precuneus**	0.0001 (0.001)	0.911	0.996	0.0001 (0.001)	0.927	0.996	-0.0012 (0.001)	0.372	0.994	-0.0008 (0.001)	0.559	0.994
**Rostral Anterior Cingulate**	-0.0002 (<0.001)	0.743	0.996	-0.0001 (<0.001)	0.790	0.996	-0.0004 (0.001)	0.506	0.994	-0.0002 (0.001)	0.710	0.994
**Rostral Middle Frontal**	-0.0004 (0.002)	0.866	0.996	0.0028 (0.003)	0.280	0.996	-0.0024 (0.003)	0.424	0.994	0.0019 (0.003)	0.583	0.994
**Superior Frontal**	0.0010 (0.003)	0.735	0.996	0.0009 (0.003)	0.758	0.996	0.0001 (0.003)	0.982	0.994	0.0003 (0.004)	0.942	0.994
**Superior Parietal**	0.0012 (0.002)	0.496	0.996	0.0003 (0.002)	0.888	0.996	0.0001 (0.002)	0.957	0.994	-0.0009 (0.002)	0.704	0.994
**Superior Temporal**	-0.0009 (0.001)	0.514	0.996	-0.0009 (0.001)	0.467	0.996	-0.0008 (0.002)	0.670	0.994	-0.0006 (0.002)	0.737	0.994
**Supramarginal**	0.0008 (0.001)	0.567	0.996	-0.0007 (0.001)	0.643	0.996	0.0006 (0.002)	0.704	0.994	-0.0003 (0.002)	0.872	0.994
**Frontal Pole**	-0.0002 (<0.001)	0.393	0.996	-0.0002 (<0.001)	0.399	0.996	-0.0001 (<0.001)	0.813	0.994	-0.0004 (<0.001)	0.214	0.994
**Temporal Pole**	0.0002 (0.000536)	0.746	0.996	0.0002 (<0.001)	0.724	0.996	-<0.0001 (0.001)	0.946	0.994	0.0001 (0.001)	0.917	0.994
**Transverse Temporal**	-0.0002 (<0.001)	0.441	0.996	-0.0002 (<0.001)	0.303	0.996	-0.0002 (<0.001)	0.500	0.994	-0.0003 (<0.001)	0.351	0.994
**Insula**	<0.001 (0.001)	0.914	0.996	-0.0004 (0.001)	0.669	0.996	-0.0001 (0.001)	0.936	0.994	-0.0006 (0.001)	0.613	0.994
**Banks of the Superior Temporal Sulcus**	-0.001 (<0.001)	0.851	0.996	-0.0007 (<0.001)	**0.025**	0.911	-0.0001 (<0.001)	0.766	0.994	-0.0011 (<0.001)	**0.006**	0.810
**Cerebellum White Matter**	-0.0010 (0.005)	0.830	0.996	0.0015 (0.003)	0.614	0.996	0.0005 (0.005)	0.914	0.994	0.0002 (0.003)	0.946	0.994
**Cerebellum Cortex**	0.0039 (0.005)	0.425	0.996	0.0003 (0.005)	0.959	0.996	0.0004 (0.006)	0.947	0.994	-0.0031 (0.007)	0.638	0.994
**Thalamus**	-0.0001 (0.001)	0.921	0.996	<0.0001 (0.001)	0.995	0.996	-0.0001 (0.001)	0.933	0.994	-0.0008 (0.001)	0.558	0.994
**Caudate**	-0.0007 (0.001)	0.211	0.996	-0.0003 (0.001)	.566	0.996	-0.0011 (0.001)	0.158	0.994	-0.0006 (0.001)	0.451	0.994
**Putamen**	-0.0011 (0.001)	0.131	0.996	0.0001 (0.0001)	0.925	0.996	-0.0016 (0.001)	0.073	0.994	0.0005 (0.001)	0.585	0.994
**Pallidum**	-0.0002 (<0.001)	0.609	0.996	-0.0005 (<0.001)	0.128	0.996	-0.0003 (<0.001)	0.590	0.994	-0.0007 (<0.001)	0.050	0.994
**Amygdala**	-<0.0001 (<0.001)	0.885	0.996	0.0001 (<0.001)	0.852	0.996	0.0002 (<0.001)	0.651	0.994	0.0001 (<0.001)	0.862	0.994

**Accumbens Area**	0.0002 (<0.001)	0.188	0.996	0.0001 (<0.001)	0.636	0.996	-<0.0001 (<0.001)	0.887	0.994	0.0001 (<0.001)	0.682	0.994
**Ventral Diencephalon**	-0.0003 (<0.001)	0.445	0.996	0.0008 (0.001)	0.163	0.996	-0.0004 (0.001)	0.523	0.994	0.0011 (0.001)	0.145	0.994
**Choroid Plexus**	-0.0008 (0.001)	0.343	0.996	-0.0001 (0.001)	0.865	0.996	-0.0012 (0.001)	0.207	0.994	0.0005 (0.001)	0.607	0.994
**Cortical White Matter**	0.0011 (0.014)	0.940	0.996	0.0111 (0.016)	0.475	0.996	-0.0078 (0.018)	0.665	0.994	-0.0021 (0.020)	0.918	0.994

Aβ, amyloid-β; FDR, false discovery rate; SE, Standard Error. **Bold** indicates statistical significance at $p < .05$ before FDR Adjustment. A negative β coefficient indicates volume/cortical atrophy over time, with strong negative coefficients indicating greater atrophy relative to the error and sample size.

Supplementary Table 4. Results of main effects of linear mixed models examining the association between change in cortical thickness and cerebral volume in those with superior cognitive performance in regions of interest both previously identified in published literature and those not previously identified.

		Left Hemisphere			Right Hemisphere		
**Brain area**	**Volume or Thickness**	**Beta (SE)**	**p**	**FDR Adjusted p**	**Beta (SE)**	**p**	**FDR Adjusted p**
**Cerebral Cortex**	Thickness	-0.0123 (0.015)	0.413	0.964	-0.0103 (0.015)	0.506	0.964
**Caudal Anterior Cingulate**	Thickness	0.0482 (0.047)	0.307	0.899	-0.0493 (0.044)	0.264	0.899
**Caudal Middle Frontal**	Thickness	-0.0251 (0.020)	0.210	0.899	0.0002 (0.022)	0.992	0.998
**Cuneus**	Thickness	-0.0004 (0.018)	0.983	0.996	-0.0015 (0.019)	0.939	0.996
**Entorhinal**	Thickness	-0.0733 (0.050)	0.145	0.786	-0.0851 (0.057)	0.139	0.786
**Fusiform**	Thickness	-0.0472 (0.022)	**0.033**	0.684	-0.0448 (0.022)	**0.044**	0.684
**Inferior Parietal**	Thickness	-0.0085 (0.018)	0.637	0.964	-0.0054 (0.019)	0.775	0.981
**Inferior Temporal**	Thickness	-0.0029 (0.025)	0.908	0.996	-0.0304 (0.025)	0.216	0.899
**Isthmus Cingulate**	Thickness	-0.0325 (0.030)	0.285	0.899	-0.0022 (0.029)	0.941	0.996
**Lateral Occipital**	Thickness	-0.0099 (0.020)	0.621	0.964	0.0072 (0.021)	0.733	0.964
**Lateral Orbitofrontal**	Thickness	0.0050 (0.021)	0.813	0.982	-0.0256 (0.025)	0.307	0.899
**Lingual**	Thickness	-0.0263 (0.017)	0.130	0.786	-0.0007 (0.018)	0.967	0.996
**Medial Orbitofrontal**	Thickness	-0.0099 (0.023)	0.665	0.964	-0.0166 (0.025)	0.511	0.964
**Middle Temporal**	Thickness	-0.0243 (0.023)	0.301	0.899	-0.0218 (0.023)	0.352	0.932
**Parahippocampal**	Thickness	-0.0236 (0.049)	0.629	0.964	-0.0446 (0.039)	0.254	0.899
**Paracentral**	Thickness	-0.0038 (0.025)	0.878	0.996	0.0082 (0.024)	0.734	0.964
**Pars Opercularis**	Thickness	-0.0039 (0.018)	0.824	0.984	0.0017 (0.019)	0.929	0.996
**Pars Orbitalis**	Thickness	>0.0001 (0.029)	0.999	0.999	-0.0394 (0.028)	0.159	0.786
**Pars Triangularis**	Thickness	-0.0229 (0.019)	0.239	0.899	-0.0152 (0.022)	0.496	0.964
**Peri Calcarine**	Thickness	-0.0062 (0.018)	0.730	0.964	-0.0005 (0.018)	0.977	0.996
**Post Central**	Thickness	-0.0066 (0.018)	0.721	0.964	-0.006 (0.019)	0.732	0.964
**Posterior Cingulate**	Thickness	0.0036 (0.026)	0.887	0.996	0.0247 (0.025)	0.329	0.899
**Precentral**	Thickness	-0.0197 (0.023)	0.386	0.964	0.0018 (0.025)	0.945	0.996
**Precuneus**	Thickness	0.0171 (0.020)	0.390	0.964	-0.0057 (0.018)	0.752	0.971
**Rostral Anterior Cingulate**	Thickness	-0.0221 (0.036)	0.538	0.964	-0.0569 (0.034)	0.095	0.684
**Rostral Middle Frontal**	Thickness	-0.0121 (0.017)	0.482	0.964	-0.0090 (0.019)	0.637	0.964
**Superior Frontal**	Thickness	-0.0052 (0.020)	0.796	0.982	0.0048 (0.020)	0.812	0.982
**Superior Parietal**	Thickness	-0.0043 (0.020)	0.828	0.984	0.0121 (0.020)	0.547	0.964
**Superior Temporal**	Thickness	-0.0375 (0.021)	0.068	0.685	-0.0343 (0.020)	0.087	0.684
**Supramargil**	Thickness	0.0025 (0.018)	0.892	0.996	-0.0061 (0.018)	0.741	0.964
**Frontal Pole**	Thickness	-0.0122 (0.035)	0.730	0.964	-0.0152 (0.040)	0.708	0.964
**Temporal Pole**	Thickness	-0.0531 (0.045)	0.242	0.899	-0.0770 (0.046)	0.096	0.684

**Transverse Temporal**	Thickness	-0.0146 (0.030)	0.620	0.964	-0.0179 (0.029)	0.541	0.964
**Insula**	Thickness	-0.0262 (0.025)	0.294	0.899	-0.0127 (0.029)	0.655	0.964
**Banks of the Superior Temporal Sulcus**	Thickness	-0.0196 (0.022)	0.378	0.964	-0.0017 (0.0244	0.945	0.996
**Cerebral Cortex**	Volume	-0.1400 (0.136)	0.305	0.899	-0.1380 (0.140)	0.327	0.899
**Caudal Anterior Cingulate**	Volume	0.0044 (0.004)	0.293	0.899	-0.0068 (0.004)	0.130	0.786
**Caudal Middle Frontal**	Volume	-0.0107 (0.009)	0.216	0.899	-0.0041 (0.009)	0.655	0.964
**Cuneus**	Volume	-0.0038 (0.004)	0.358	0.932	-0.0020 (0.004)	0.618	0.964
**Entorhinal**	Volume	-0.0037 (0.003)	0.240	0.899	-0.0024 (0.003)	0.494	0.964
**Fusiform**	Volume	-0.0198 (0.012)	0.093	0.685	-0.0279 (0.011)	0.009	0.601
**Inferior Parietal**	Volume	-0.0243 (0.013)	0.070	0.685	-0.0100 (0.015)	0.493	0.964
**Inferior Temporal**	Volume	-0.0050 (0.012)	0.721	0.964	-0.0146 (0.0139)	0.261	0.899
**Isthmus Cingulate**	Volume	0.0061 (0.004)	0.092	0.684	-0.0005 (0.004)	0.893	0.995
**Lateral Occipital**	Volume	-0.0008 (0.011)	0.947	0.996	0.0003 (0.012)	0.980	0.996
**Lateral Orbitofrontal**	Volume	-0.0036 (0.007)	0.598	0.964	-0.0064 (0.006)	0.327	0.899
**Lingual**	Volume	-0.0037 (0.008)	0.657	0.964	-0.0035 (0.007)	0.632	0.964
**Medial Orbitofrontal**	Volume	-0.0072 (0.005)	0.162	0.786	0.0020 (0.005)	0.702	0.964
**Middle Temporal**	Volume	0.0033 (0.012)	0.779	0.981	0.0007 (0.012)	0.958	0.996
**Parahippocampal**	Volume	>0.0001 (0.003)	0.984	0.996	-0.0014 (0.003)	0.650	0.964
**Paracentral**	Volume	-0.0059 (0.004)	0.151	0.786	-0.0034 (0.005)	0.478	0.964
**Pars Opercularis**	Volume	0.0011 (0.006)	0.846	0.996	-0.0036 (0.005)	0.501	0.964
**Pars Orbitalis**	Volume	0.0004 (0.003)	0.869	0.996	-0.0058 (0.003)	0.088	0.684
**Pars Triangularis**	Volume	-0.0054 (0.005)	0.255	0.899	-0.0020 (0.005)	0.714	0.964
**Peri Calcarine**	Volume	-0.0012 (0.003)	0.734	0.964	0.0003 (0.004)	0.939	0.996
**Post Central**	Volume	-0.0080 (0.010)	0.407	0.964	0.0065 (0.010)	0.530	0.964
**Posterior Cingulate**	Volume	0.0002 (0.004)	0.946	0.996	-0.0051 (0.004)	0.163	0.786
**Precentral**	Volume	-0.0238 (0.013)	0.068	0.685	-0.0047 (0.013)	0.722	0.964
**Precuneus**	Volume	0.0033 (0.008)	0.698	0.964	-0.0022 (0.009)	0.803	0.982
**Rostral Anterior Cingulate**	Volume	0.0017 (0.004)	0.678	0.964	-0.0142 (0.004)	**0.001**	0.164
**Rostral Middle Frontal**	Volume	-0.0023 (0.0144)	0.872	0.996	-0.0083 (0.015)	0.581	0.964
**Superior Frontal**	Volume	-0.0076 (0.018)	0.675	0.964	-0.0044 (0.019)	0.814	0.982
**Superior Parietal**	Volume	0.0061 (0.0144)	0.671	0.964	0.0191 (0.013)	0.142	0.786
**Superior Temporal**	Volume	-0.0117 (0.0103)	0.258	0.899	-0.0061 (0.010)	0.545	0.964
**Supramargil**	Volume	-0.0027 (0.0103)	0.796	0.982	-0.0066 (0.0114)	0.565	0.964
**Frontal Pole**	Volume	-0.0012 (0.001)	0.424	0.964	-0.0007 (0.002)	0.719	0.964
**Temporal Pole**	Volume	-0.0023 (0.004)	0.571	0.964	-0.0042 (0.004)	0.307	0.899
**Transverse Temporal**	Volume	-0.0003 (0.002)	0.878	0.996	-0.0006 (0.001)	0.675	0.964
**Insula**	Volume	-0.0065 (0.007)	0.320	0.899	-0.0130 (0.007)	0.053	0.685

**Banks of the Superior Temporal Sulcus**	Volume	0.0012 (0.004)	0.741	0.964	-0.0034 (0.003)	0.287	0.899
**Cerebellum White Matter**	Volume	0.0505 (0.022)	**0.021**	0.684	0.0309 (0.020)	0.129	0.786
**Cerebellum Cortex**	Volume	0.0450 (0.060)	0.453	0.964	0.0218 (0.057)	0.701	0.964
**Thalamus Proper**	Volume	0.0161 (0.008)	**0.038**	0.684	0.0049 (0.006)	0.415	0.964
**Caudate**	Volume	-0.0013 (0.005)	0.792	0.981	0.0024 (0.005)	0.663	0.964
**Putamen**	Volume	-0.0140 (0.007)	**0.039**	0.684	-0.0056 (0.006)	0.356	0.932
**Pallidum**	Volume	-0.0037 (0.003)	0.222	0.899	0.0043 (0.002)	**0.030**	0.685
**Hippocampus**	Volume	0.0062 (0.005)	0.175	0.820	0.0026 (0.005)	0.582	0.964
**Amygdala**	Volume	0.0012 (0.002)	0.578	0.964	-0.0011 (0.002)	0.642	0.964
**Accumbens Area**	Volume	-0.00211 (0.001)	0.079	0.684	-0.0017 (0.001)	0.148	0.786
**Ventral Diencephalon**	Volume	0.0015 (0.003)	0.634	0.964	-0.0003 (0.003)	0.908	0.996
**Choroid Plexus**	Volume	0.0081 (0.003)	**0.011**	0.601	0.0088 (0.004)	**0.023**	0.685
**Cortical White Matter**	Volume	0.267 (0.141)	0.061	0.684	0.2520 (0.145)	0.084	0.685

FDR, false discovery rate; SE, Standard Error. **Bold** indicates statistical significance at $p < 0.05$ before FDR Adjustment.
A negative β coefficient indicates volume/cortical atrophy over time, with strong negative coefficients indicating greater atrophy relative to the error and sample size.
